# Tumor Typing of Endocervical Adenocarcinoma: Contemporary Review and Recommendations From the International Society of Gynecological Pathologists

**DOI:** 10.1097/PGP.0000000000000751

**Published:** 2021-02-09

**Authors:** Simona Stolnicu, Kay J. Park, Takako Kiyokawa, Esther Oliva, W. Glenn McCluggage, Robert A. Soslow

**Affiliations:** Department of Pathology, University of Medicine, Pharmacy, Sciences and Technology of Targu Mures, Romania (S.S.); Department of Pathology, Memorial Sloan Kettering Cancer Center, New York, New York (K.J.P., R.S.S.); Department of Pathology, Jikei University School of Medicine, Tokyo, Japan (T.K.); Department of Pathology, Massachusetts General Hospital, Boston, Massachusetts (S.O.); Department of Pathology, Belfast Health and Social Care Trust, Belfast, UK (G.M.C.)

**Keywords:** Endocervical adenocarcinoma, Cervical cancer, HPV-associated, HPV-independent, ISGyP, Types, Classification

## Abstract

The incidence of endocervical adenocarcinoma, the second most common cervical cancer in the world, has been on the rise. While most cervical cancers are squamous cell carcinomas and associated with high-risk oncogenic human papillomavirus (HPV), approximately 15% of endocervical adenocarcinomas, which now represent about one quarter of all cervical cancers, are HPV-independent. In this review, we will focus on the shortcomings of historical histologic classification systems of female genital tract tumors as they pertain to endocervical adenocarcinomas, and we will highlight the advantages of the new International Endocervical Adenocarcinoma Criteria and Classification system, which forms the basis for the WHO 2020 classification. We will cover the various histologic types, subtypes, and variants of endocervical adenocarcinoma with regard to morphology, immunophenotype, molecular genetics, HPV status and differential diagnosis, and we will provide International Society of Gynecological Pathologists recommendations for diagnosing these tumors.

Endocervical adenocarcinomas are a heterogeneous group of neoplasms, contrary to what was previously thought. Screening strategies designed for and effective in detecting squamous cell carcinoma precursors are less effective in detecting endocervical glandular precursor lesions. Because of this and other reasons, the real and relative incidence of endocervical adenocarcinoma has increased in recent years from 5% to up to 20-25%, particularly in patients 30 yr of age or older, according to studies from the United States and Europe 1–4. Most endocervical adenocarcinomas are associated with high-risk oncogenic human papillomavirus (HPV) most commonly HPV 18, 16, and 45 5–7. Unlike squamous cell carcinoma of the cervix, however, ~15% of all endocervical adenocarcinomas are not associated with HPV (HPV-independent) 8–13 and harbor distinct molecular alterations. This fact, along with the shortcomings of the 2004 World Health Organization (WHO) Classification, prompted changes to the new 2020 WHO categorization of endocervical adenocarcinomas, which is the subject of this review 14. Recommendations for reporting are provided based on the 2020 WHO Classification, a literature review, and the opinion of the International Society of Gynecological Pathologists (ISGyP) working group on endocervical adenocarcinomas.

## HISTOLOGIC CLASSIFICATION OF ENDOCERVICAL ADENOCARCINOMA

### Shortcomings of Previous Classification Schemes

Endocervical adenocarcinomas are morphologically heterogeneous and have traditionally been diagnosed based on morphology, primarily on tumor architecture and the presence of intracytoplasmic mucin (WHO 2014 classification system). The diagnosis of many tumor types included in the WHO 2014 classification were poorly reproducible 15, with very little clinical relevance. For example, the National Comprehensive Cancer Network (NCCN) and European Society of Gynaecological Oncology (ESGO) guidelines recommend that endocervical adenocarcinomas be managed based on stage and other tumor-related factors, such as tumor size and lymphovascular space invasion, rather than histotype, despite variations in morphology, etiology, and clinical behavior. The exception to these guidelines concerns fertility preservation, which is not recommended for gastric-type adenocarcinomas and neuroendocrine carcinomas 16,17.

The previous WHO 2014 classification categorized endocervical adenocarcinomas into the following types: usual, mucinous, villoglandular, clear cell, serous, endometrioid, and mesonephric 5. Issues with this classification system, however, include the following:

1. Usual-type endocervical adenocarcinoma was defined as “the most common type of adenocarcinoma with relative mucin depletion” but without any specific defining or quantitative criteria.
2. Endometrioid endocervical adenocarcinoma was defined as an adenocarcinoma that morphologically resembled endometrial endometrioid adenocarcinomas, just like mucin-depleted usual types, but there are no criteria for differentiating usual-type from endometrioid-type endocervical adenocarcinomas based on morphology. Subsequent studies, discussed later in the text, have shown that this is an extraordinarily rare type of primary endocervical adenocarcinoma 13.
3. Mucinous endocervical adenocarcinomas included gastric-type, intestinal-type, and signet ring cell-type tumors, which is problematic as tumors of various etiologies (HPV-associated or HPV-independent) can be present within these groups. For example, adenocarcinomas with goblet cells (intestinal) can be either HPV-associated or HPV-independent. In addition, it is now well established that gastric-type mucinous adenocarcinomas is a highly aggressive tumor etiologically unrelated to HPV infection, yet this is not taken into account in the classification of these tumors 18,19.
4. Serous endocervical adenocarcinomas were defined as morphologically identical to endometrial or adnexal serous carcinomas, yet the WHO 2014 classification system states that they can be HPV-positive “serous-like” in younger patients and *TP53* mutant in older patients, effectively combining 2 seemingly different tumors into a single category. Finally, serous carcinoma is currently not categorized as a type of primary endocervical adenocarcinoma.

### The International Endocervical Adenocarcinoma Criteria and Classification (IECC) System for Endocervical Adenocarcinomas and the WHO 2020 Classification

The IECC, which was first described in 2018, forms the basis for the WHO 2020 classification 14, which divides endocervical adenocarcinomas into different types based on HPV association. The IECC correlates with clinical features, p16 expression and HPV status, prognostic parameters, survival, and response to treatment 13,19,20. Compared with the WHO 2014 system, the IECC has shown superior interobserver agreement among gynecologic pathologists and a highly significant correlation with HPV status, suggesting it is a more biologically significant and clinically valuable system for classifying endocervical adenocarcinomas 15. Note that there are very rare endocervical adenocarcinomas that cannot be classified morphologically [adenocarcinoma not otherwise specified (NOS)], and these may be either HPV-associated or HPV-independent, unlike the other tumor types. The tumor types as delineated by the WHO 2014 and IECC/WHO 2020 systems are summarized in Table 1.

**TABLE 1:** Endocervical adenocarcinoma classifications according to the WHO 2014 and IECC 2018/WHO 2020 classification systems

WHO 2014	IECC 2018/WHO 2020
Endocervical adenocarcinoma, usual type	HPV-associated endocervical adenocarcinoma
Mucinous carcinoma NOS	Usual type
Mucinous carcinoma, gastric type	Mucinous (NOS, intestinal, signet ring cell, ISMC)
Mucinous carcinoma, intestinal type	Adenocarcinoma NOS
Mucinous carcinoma, signet-ring cell type	HPV-independent endocervical adenocarcinoma
Villoglandular carcinoma	Gastric type
Mesonephric carcinoma	Mesonephric type
Serous carcinoma	Endometrioid type
Clear cell carcinoma	Clear cell type
Endometrioid carcinoma	Adenocarcinoma NOS
Adenocarcinoma NOS	

HPV indicates human papillomavirus; IECC, International Endocervical Adenocarcinoma Criteria and Classification; ISMC, invasive stratified mucinous carcinoma; NOS, not otherwise specified; WHO, World Health Organization.

The IECC system is based on identifying mitotic figures and apoptotic bodies at 40 to 100× magnification on hematoxylin & eosin (H&E)-stained slides and, therefore, is easy to apply in daily practice. When easily identifiable mitoses and apoptotic bodies are seen at this power, the tumor is likely HPV-associated; when mitoses and apoptotic bodies are absent or found with difficulty at high magnification, the tumor is likely HPV-independent 13,21. HPV-associated precursor lesions include usual-type endocervical adenocarcinoma in situ (AIS) and stratified mucin-producing intraepithelial lesions. HPV-independent precursor lesions are recognized for gastric-type adenocarcinomas, namely atypical lobular endocervical glandular hyperplasia and gastric AIS (gAIS) 22–25, and some mesonephric carcinomas (also HPV-independent) likely arise in the setting of mesonephric hyperplasia. HPV-associated and HPV-independent endocervical adenocarcinomas are subsequently classified based on the obvious presence of intracytoplasmic mucin and architectural patterns such as cellular stratification (for HPV-associated mucinous carcinomas, for example) and existing criteria (for HPV-independent tumors). The classification system also includes newly described histologic variants such as invasive stratified mucinous carcinoma (ISMC) and those with micropapillary features (micropapillary adenocarcinoma) 22,26,27. The main advantage of this new classification system is the ease of its application in routine practice in any laboratory across the world, as it generally only requires H&E slides without the need for expensive and sophisticated additional tests.

### Recommendations Regarding the Classification of Endocervical Adenocarcinomas

A recent workshop dedicated to endocervical adenocarcinoma was organized by the ISGyP in Los Angeles before the 2020 United States and Canadian Academy of Pathology (USCAP) annual meeting. Key points and recommendations from the workshop included the following:

- Endocervical adenocarcinomas should be classified according to the forthcoming WHO 2020 classification system, which incorporates the IECC system.
- Both classification systems categorize endocervical adenocarcinomas into HPV-associated and HPV-independent types using morphology alone.
- The WHO 2020 and IECC systems includes newly described microscopic variants of HPV-associated endocervical adenocarcinomas.
- Ancillary testing for diagnosis (such as p16) does not need to be reflexively performed, as the morphology is tightly linked to HPV status. p16 and high-risk HPV testing should be reserved for difficult or ambiguous cases, such as the occasional HPV-associated adenocarcinoma lacking easily identifiable mitoses and apoptotic bodies.
- If interpretation is difficult, a diagnostic algorithm based on the amount of intracytoplasmic mucin and ancillary testing (such as HPV testing, p16, and GATA3 immunohistochemistry) may be useful in the differential diagnosis of the various histologic types and histologic mimics 28.
- RNA-based in situ hybridization for high-risk HPV exhibits higher sensitivity and specificity compared with HPV DNA polymerase chain reaction (PCR) 29.
- RNA-based in situ hybridization for high-risk HPV, although not available in most institutions, may have superior sensitivity, specificity, and positive and negative predictive values compared with p16 in identifying HPV-associated endocervical adenocarcinomas 13.

## HPV-ASSOCIATED ENDOCERVICAL ADENOCARCINOMA

HPV-associated endocervical adenocarcinomas include the usual type, mucinous type, and HPV-associated adenocarcinoma NOS. These tumor types may arise from an in situ component, such as usual-type endocervical AIS and stratified mucin-producing intraepithelial lesion, but this is not seen in every case.

### Usual-type Endocervical Adenocarcinomas (Including Villoglandular and Micropapillary Architectural Variants)

The usual type is the most common HPV-associated endocervical adenocarcinoma, accounting for 75% to 80% of all endocervical adenocarcinomas 13. This type was previously referred to as the endocervical type with obvious cytoplasmic mucin and endometrioid type when mucin-depleted. The IECC defines the usual subtype as a tumor composed of up to 50% of tumor cells with appreciable intracytoplasmic mucin assessed on H&E-stained slides 13.

### Issues Regarding Usual-type Endocervical Adenocarcinomas

- When lacking intracytoplasmic mucin, a “pseudoendometrioid” morphology can pose problems in the differential diagnosis with endometrioid adenocarcinoma of the cervix or corpus.
- Villoglandular and micropapillary architectural patterns are variants of usual-type tumors and may be associated with more typical areas.
- They may have a “serous-like” morphology, and therefore, metastatic serous carcinomas from other organs may be considered.
- They rarely contain benign-appearing squamous elements, making it difficult to distinguish them from adenosquamous carcinomas or endometrioid adenocarcinomas with squamous differentiation of the corpus.

**FIG. 1:**
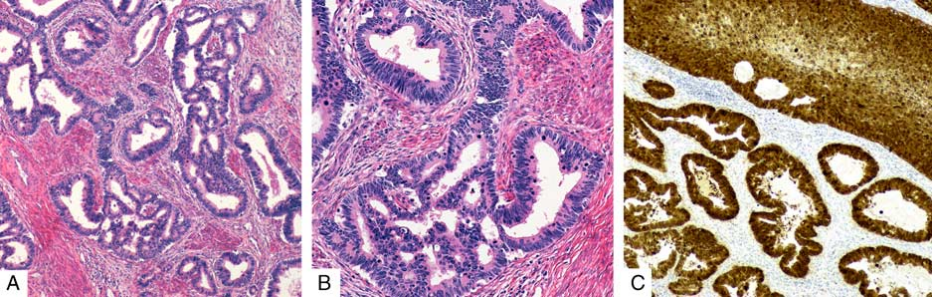
Usual-type endocervical adenocarcinoma, human papillomavirus–associated. Note the apical mitotic figures and apoptotic bodies (A, B), and block-type p16 staining in adenocarcinoma and overlying high-grade squamous intraepithelial lesion (C).

Characteristically, the tumor cells of usual-type endocervical adenocarcinomas are columnar, with pseudostratified elongated and hyperchromatic nuclei. The cytoplasm is usually mucin-depleted, and the presence of apical mitotic figures and basal apoptotic bodies is virtually pathognomonic (although not seen in every case). Intracytoplasmic mucin can still be seen, sometimes extensively within parts of a tumor, although they do not reach the 50% threshold for mucinous-type tumors. In rare cases, the presence of benign squamous metaplasia, particularly when extensive, can mimic adenosquamous carcinomas or endometrioid adenocarcinomas with squamous differentiation. The stroma is often, but not always desmoplastic, and sometimes there is an accompanying inflammatory infiltrate, necrosis, or pools of mucin (Fig. 1).

**FIG. 2:**
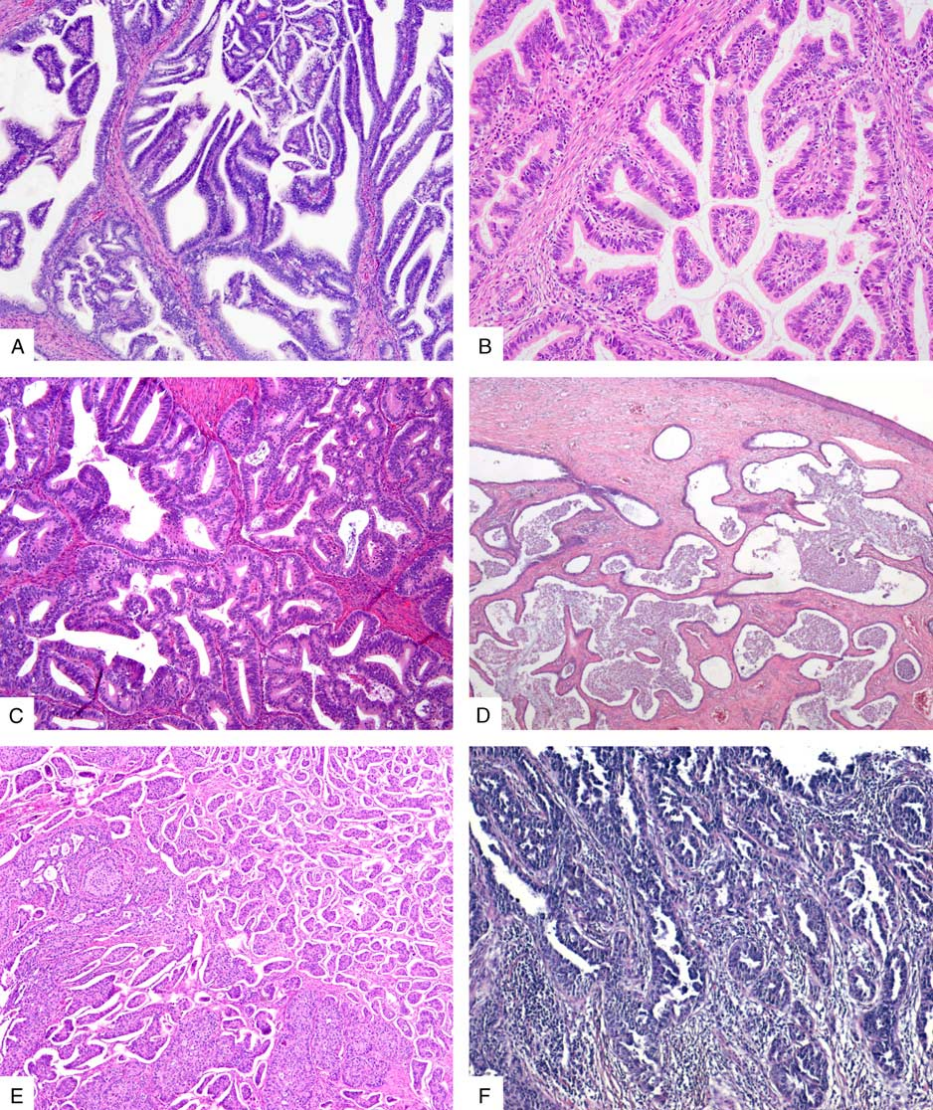
Usual-type adenocarcinoma, human papillomavirus–associated, variant patterns: (A) villoglandular, (B) papillary, (C) cribriform, resembling “endometrioid carcinoma,” (D) cystic glands, (E) serous-like (micropapillary), and (F) serous-like (papillary and micropapillary with high nuclear grade and ragged luminal contours).

Architecturally, usual-type endocervical adenocarcinomas are predominantly composed of glands with irregular shapes and sizes and smooth luminal borders. Papillary, cribriform, and solid patterns can also occur. Uncommonly, villoglandular, micropapillary, macrocystic, microcystic, trabecular, and single-cell patterns (mimicking breast lobular carcinoma) can occur (Fig. 2) 30.

Villoglandular architecture deserves special mention, as it had been previously considered a distinct type of endocervical adenocarcinoma with an excellent prognosis 5,31. Villoglandular adenocarcinoma is characterized by prominent exophytic papillary growth in the superficial portion of the tumor, composed of papillae of variable thickness and length containing central fibrous cores and lined by columnar pseudostratified epithelial cells that exhibit low-grade nuclear atypia. Purely exophytic growth associated with villoglandular architecture is uncommon. When stromal invasion is present at the base of the villoglandular proliferation, it tends to resemble HPV-associated usual-type adenocarcinoma. Even when purely villoglandular, the tumor is always HPV-positive, with characteristic apical mitoses and apoptotic bodies and a immunohistochemical profile similar to that of usual-type tumors. Therefore, we consider these tumors usual-type endocervical adenocarcinoma variants rather than separate tumor types. As such, when this growth pattern is prominent, we recommend calling these tumors usual-type HPV-associated endocervical adenocarcinomas with villoglandular architecture.

A micropapillary pattern, occurring alone or admixed with other HPV-associated patterns, can also be seen in HPV-associated endocervical adenocarcinomas (especially usual type). This recently recognized architectural variant is microscopically represented by small, tightly cohesive papillary groups of neoplastic cells with eosinophilic cytoplasm and atypical nuclei, typically surrounded by clear spaces resembling vascular channels 26. This architectural pattern is very commonly associated with lymphovascular space invasion, lymph node involvement, and a poor prognosis. Therefore, it is important that the pathologist recognize this pattern and include it in the pathology report.

Usual-type tumors almost always exhibit diffuse p16 positivity (defined as block-type positivity, with nuclear and/or cytoplasmic staining of essentially every tumor cell) and are positive for HPV by RNA in situ hybridization, the most sensitive method for detecting high-risk HPV-associated types 13. Anything less than block-type immunoreactivity with p16 is not supportive of a high-risk HPV-associated adenocarcinoma. Of note, both HPV in situ hybridization and p16 can be negative (defined as completely p16 negative or patchy non–block-type immunoreactivity) in poorly fixed tissues and older paraffin blocks. The typical immunoprofile of usual-type HPV-associated adenocarcinoma is negative staining for estrogen receptor (ER), progesterone receptor (PR), vimentin, MUC6, HNF1beta, Napsin A, GATA3, androgen receptor (AR), and human epidermal growth factor receptor 2 (HER2); p53 is usually wild-type. However, these markers can sometimes be positive/aberrant; for example, it is not uncommon for ER to be focally and weakly positive 28. These findings are especially important when differentiating usual-type HPV-associated tumors from other endocervical adenocarcinoma types or extra-cervical tumors infiltrating the cervix.

An important differential diagnostic consideration for usual-type HPV-associated endocervical adenocarcinoma is endometrioid-type adenocarcinoma of the cervix or corpus. Cervical endometrioid adenocarcinoma, discussed later, is very rare, thought to arise in the setting of endometriosis, and is HPV-negative. As a rule, mucin-depleted invasive adenocarcinomas of the cervix should not be diagnosed as endometrioid type in the presence of prominent apical mitotic figures and apoptotic bodies, as these lesions are almost invariably mucin-depleted usual-type HPV-associated adenocarcinomas. Distinguishing HPV-associated adenocarcinomas from endometrial endometrioid adenocarcinomas is particularly important, as clinical management differs. This differential diagnosis can be complicated in biopsy material and curettings, which often contain limited material. ER, PR, and vimentin are generally positive in endometrioid adenocarcinomas of the endometrium, whereas p16 is patchy/negative and HPV is negative. These markers should be interpreted with caution, as high-grade endometrioid adenocarcinomas of the corpus can be negative for ER and PR and rarely p16 block-positive. Furthermore, usual-type HPV-associated tumors of the cervix can uncommonly be ER, PR, and vimentin-positive, and very rarely p16-negative. Loss of one or more mismatch repair (MMR) proteins (MLH1, PMS2, MSH2, and MSH6) in tumor cells is strongly suggestive of an endometrioid adenocarcinoma of the corpus with microsatellite instability. CEA has limited diagnostic usefulness, as focal staining can be observed in both tumors. In difficult cases, mRNA high-risk HPV in situ hybridization, together with clinical features, can be diagnostically helpful.

Usual-type HPV-associated endocervical adenocarcinomas with papillary architecture and high-grade nuclear features (including the micropapillary variant) can also mimic serous carcinoma of the cervix, and the differential diagnosis may include direct involvement or a drop metastasis of tubo-ovarian or endometrial serous carcinoma. Serous carcinoma of the cervix is no longer accepted as a primary tumor type, as all such tumors are p16-negative and HPV-positive, and almost always p53 wild-type, in contrast to true serous carcinomas such tumors are regarded as HPV-associated endocervical adenocarcinomas rather than serous carcinomas.

Recent studies have suggested that it may be important to differentiate between usual- and mucinous-type HPV-associated endocervical adenocarcinomas due to possible differences in clinical behavior and survival outcomes (mucinous-type tumors are associated with worse outcomes); this should be confirmed by future study of additional cohorts 20,32,33. The IECC-defined threshold (greater than 50% of tumor cells with intracytoplasmic mucin) should be used for a mucinous-type diagnosis.

Prevalent molecular alterations in usual-type HPV-associated endocervical adenocarcinomas include mutations in *PIK3CA*, *KRAS*, and *PTEN*, and abnormalities in segments of the PI3K/Akt/mTOR signaling cascade, some of which have predictive and prognostic value 34–37.

### Mucinous-type Endocervical Carcinomas (Including Mucinous NOS, Intestinal, Signet-ring, and ISMC Variants)

The WHO 2014 classification system grouped HPV-positive and HPV-negative tumors (such as gastric-type adenocarcinomas) with abundant intracytoplasmic mucin in the mucinous category. The IECC and WHO 2020 systems, however, include mucinous (non–gastric-type) adenocarcinoma as an HPV-associated adenocarcinoma, with the following subcategories:

- Mucinous NOS: ≥50% of tumor cells have intracytoplasmic mucin in a usual-type background.
- Intestinal: goblet cells, representing ≥50% of cells in a usual-type or mucinous background. Paneth-like cells can also be seen occasionally.
- Signet-ring: round cells with a mucinous vacuole that displaces the nucleus to the periphery, representing ≥50% of cells in a usual-type background.
- ISMC: invasive nests of stratified columnar cells with variable amounts of intracytoplasmic mucin and peripheral palisading 13.

**FIG. 3:**
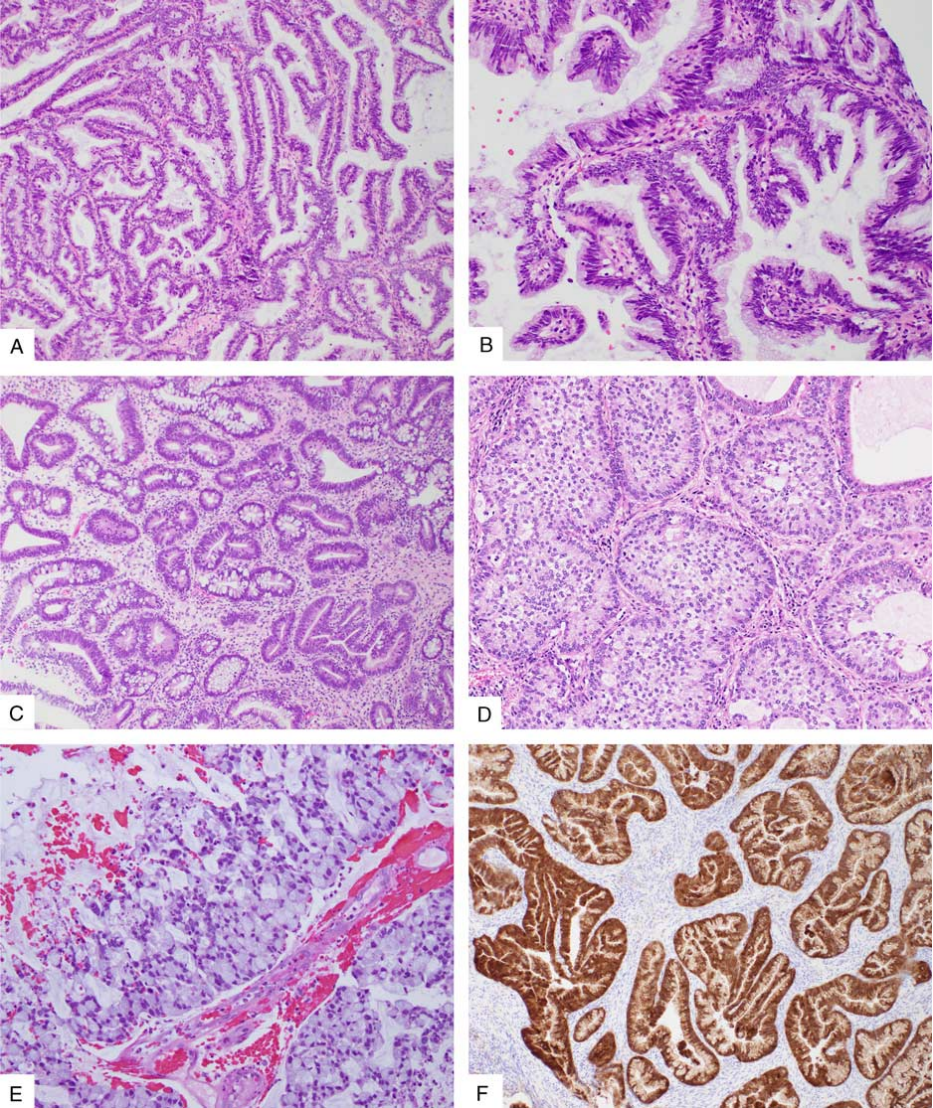
Mucinous adenocarcinoma, human papillomavirus–associated. The not otherwise specified pattern resembles usual-type adenocarcinoma, but it contains obvious cytoplasmic mucin (A, B), (C) intestinal-type mucinous differentiation, (D) invasive stratified mucinous carcinoma, (E) signet ring cell adenocarcinoma, and (F) block-like p16 staining.

These tumors, which are not often encountered in routine practice, can display various architectures, such as glandular, insular, solid, trabecular, and single-cell patterns. As they are all HPV-associated, they pathognomonically display mitotic activity and apoptotic bodies at 40 to 100× magnification. The stroma can display mucin pools and inflammatory infiltrates (Fig. 3).

### Issues Regarding Mucinous-type Endocervical Adenocarcinomas

- All subtypes can display a wide range of architectural and cytologic diversity.
- The signet-ring subtype is very rare and, if pure, may indicate metastasis to the cervix.
- The signet-ring subtype may be admixed with usual-type.
- Intestinal differentiation is also found in gastric-type tumors, which are HPV-independent (see below).
- Because of the intracytoplasmic mucin, mucinous-type can be misdiagnosed as gastric-type adenocarcinomas, endometrioid adenocarcinomas with mucinous differentiation, and metastases from other organs.
- ISMC can occur in pure form or admixed with usual-type or mucinous-type HPV-associated endocervical adenocarcinomas, adenosquamous, or neuroendocrine carcinomas.

The recently described ISMC type of HPV-associated mucinous endocervical adenocarcinoma has been reported to have a poorer prognosis than that of other HPV-associated types 27,32,33. ISMC can occur in pure form or it can be associated with usual or mucinous differentiation, and as adenosquamous or neuroendocrine carcinoma. ISMC displays a wide range of architectural diversity, including insular, glandular, solid, papillary, trabecular, micropapillary, and single-cell formations 38. Their cytologic appearance can also vary, with variable amounts of mucin (mucin-rich to mucin-poor), cytoplasmic clearing, histiocytoid-like features, “glassy cell”–like features, signet-ring–like features, bizarre nuclear atypia, “squamoid differentiation” in the form of cells with dense eosinophilic cytoplasm lacking intercellular bridges, and keratinization. Intraepithelial neutrophilic infiltrates, apoptotic bodies, and frequent mitotic figures are commonly easily identified. These features are important to recognize when making a diagnosis of ISMC, as it can mimic other tumor subtypes 27,38.

Essentially all mucinous-type HPV-associated endocervical adenocarcinomas exhibit block-type positivity for p16 and HPV positivity 13. These neoplasms are usually negative for ER, PR, vimentin, MUC6, GATA3, and CK20, but can be positive for CAIX, HNF1beta, and Napsin A. The intestinal subtype can be positive for CDX2 and CK20 28,39. ISMC can be focally positive with p63 and p40, especially at the periphery of cell nests 40. Unlike other HPV-associated tumors, ISMCs may show mutation-type p53 staining, and less frequently, PAX8 labeling 28,41.

Various HPV-associated mucinous-type endocervical adenocarcinomas are sometimes misdiagnosed as gastric-type endocervical adenocarcinoma, and the distinction may be particularly difficult with a small biopsy specimen. Most HPV-associated mucinous-type tumors have easily detectable mitotic figures and apoptotic bodies at 40 to 100× magnification, whereas mitoses and apoptotic bodies are typically rare in gastric-type tumors. Intestinal differentiation can occur in both types. p16 and HPV testing can assist with diagnosis in problematic cases, but p16 is rarely negative in HPV-associated carcinomas and rarely positive in gastric type carcinomas.

In addition, HPV-associated mucinous-type endocervical adenocarcinomas can be misdiagnosed as endometrioid adenocarcinoma of the endometrium with extensive mucinous differentiation, and an algorithm for diagnosis has been proposed for tumors with abundant intracytoplasmic mucin using HPV testing and ER immunohistochemistry 13. Furthermore, p16 can be used to differentiate the 2 tumor types, and it is more readily available than HPV testing. Finally, metastasis from another organ should be ruled out in tumors with abundant intracytoplasmic mucin, and clinically relevant information together with ancillary tests, including a panel of immunohistochemical stains and HPV testing, can be used.

### HPV-associated Adenocarcinoma and NOS

**FIG. 4:**
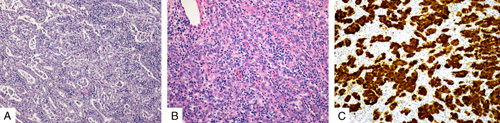
Adenocarcinoma, not otherwise specified (NOS), either human papillomavirus (HPV)-independent (A) or HPV-associated (B, C). (C) Block-type staining for p16 confirms HPV association of the carcinoma illustrated in panel B. p16 staining in adenocarcinoma, NOS, HPV-independent (A) was patchy, non–block-type (not shown).

This very rare tumor is HPV-associated but cannot be classified morphologically into any of the known categories (Fig. 4B, C). Of note, this diagnostic category should be used sparingly. Microscopically, it is usually predominantly solid, with high-grade nuclear atypia and minimal intracytoplasmic mucin. Mitotic figures and apoptotic bodies are typically easily identified. This tumor exhibits block-type positivity for p16 and is HPV-positive. The differential diagnoses include other HPV-associated and HPV-independent endocervical adenocarcinoma types, and poorly differentiated squamous cell carcinomas. The latter is usually p63-positive and p40-positive, whereas these markers are negative in HPV-associated adenocarcinoma NOS. Adenocarcinoma NOS-type can also mimic adenosquamous carcinoma, but in the latter tumor, 2 different malignant components, squamous and glandular, should be recognized on H&E-stained sections.

### Recommendations for the Diagnosis of HPV-associated Endocervical Adenocarcinomas

- Usual-type tumors lacking intracytoplasmatic mucin should not be diagnosed as endometrioid-type tumors.
- HPV-associated endocervical adenocarcinomas with villoglandular and micropapillary patterns can be designated as usual-type tumors, but these patterns should be noted on the pathology report.
- A diagnosis of primary cervical serous carcinoma should not be made when dealing with serous-like morphology; most examples will represent an HPV-associated endocervical adenocarcinoma with serous-like morphology or a metastasis from the uterine corpus or fallopian tube/ovary.
- A micropapillary component of any percentage has a propensity for aggressive behavior and should be reported.
- Similarly, ISMC of any percentage has a propensity for aggressive behaviour and should be reported.
- Mucinous-type (including NOS) tumors are likely associated with a worse survival compared with usual-type tumors, so keeping these 2 categories distinct and reporting them separately is recommended until more studies are conducted.
- Whereas p16 and HPV testing are not always needed, they may be useful in problematic cases, and in the absence of block-type p16 staining or HPV, a diagnosis of HPV-associated endocervical adenocarcinoma should be questioned.

## HPV-INDEPENDENT ENDOCERVICAL ADENOCARCINOMAS

HPV-independent endocervical adenocarcinomas account for ~15% of endocervical adenocarcinomas in most Western countries (the incidence may be higher in East Asia due to a relatively high frequency gastric-type tumors in this region). These tumors include gastric, clear cell, mesonephric and endometrioid-type carcinomas and adenocarcinoma NOS. Preinvasive premalignant lesions associated with gastric-type endocervical adenocarcinoma are lobular endocervical glandular hyperplasia (LEGH), specifically atypical LEGH and gastric-type adenocarcinoma in situ. There are no identified precursor lesions for clear cell and endometrioid-type tumors 23–25,41,42.

### Gastric-type Endocervical Adenocarcinoma

Gastric-type endocervical adenocarcinoma, initially described by Kojima and colleagues, is the second most common adenocarcinoma of the cervix, accounting for >20% of all cervical adenocarcinomas in Japan 13,43,44. Gastric-type endocervical adenocarcinoma is defined as a tumor containing cells displaying gastric differentiation with abundant clear or pale eosinophilic cytoplasm, distinct cytoplasmic borders, generally low nuclear to cytoplasmic ratio, and basally located nuclei. Mitotic figures and apoptosis are present but are typically inconspicuous and not detected easily at 40 to 100× magnification 13. Precursor lesions include atypical LEGH and gAIS, the latter only recently described 24,25. Very rare gastric-type mucinous adenocarcinomas of the vagina and endometrium have been reported 45,46.

### Issues Regarding Gastric-type Endocervical Adenocarcinoma

- The minimal deviation pattern (previously known as adenoma malignum) can be difficult to diagnose and differentiate from a benign glandular lesion, especially with a small biopsy specimen.
- Gastric-type tumors have a morphology and immunohistochemical profile similar to pancreatic ductal adenocarcinoma and cholangiocarcinoma.
- Gastric-type tumors can have variable architectures and cytologic features mimicking other neoplasms.
- There are currently no totally reliable “positive” markers to establish a diagnosis of gastric-type endocervical adenocarcinoma.
- p16 is usually negative or focally positive but can be diffusely positive (block-type) in a minority of cases.

**FIG. 5:**
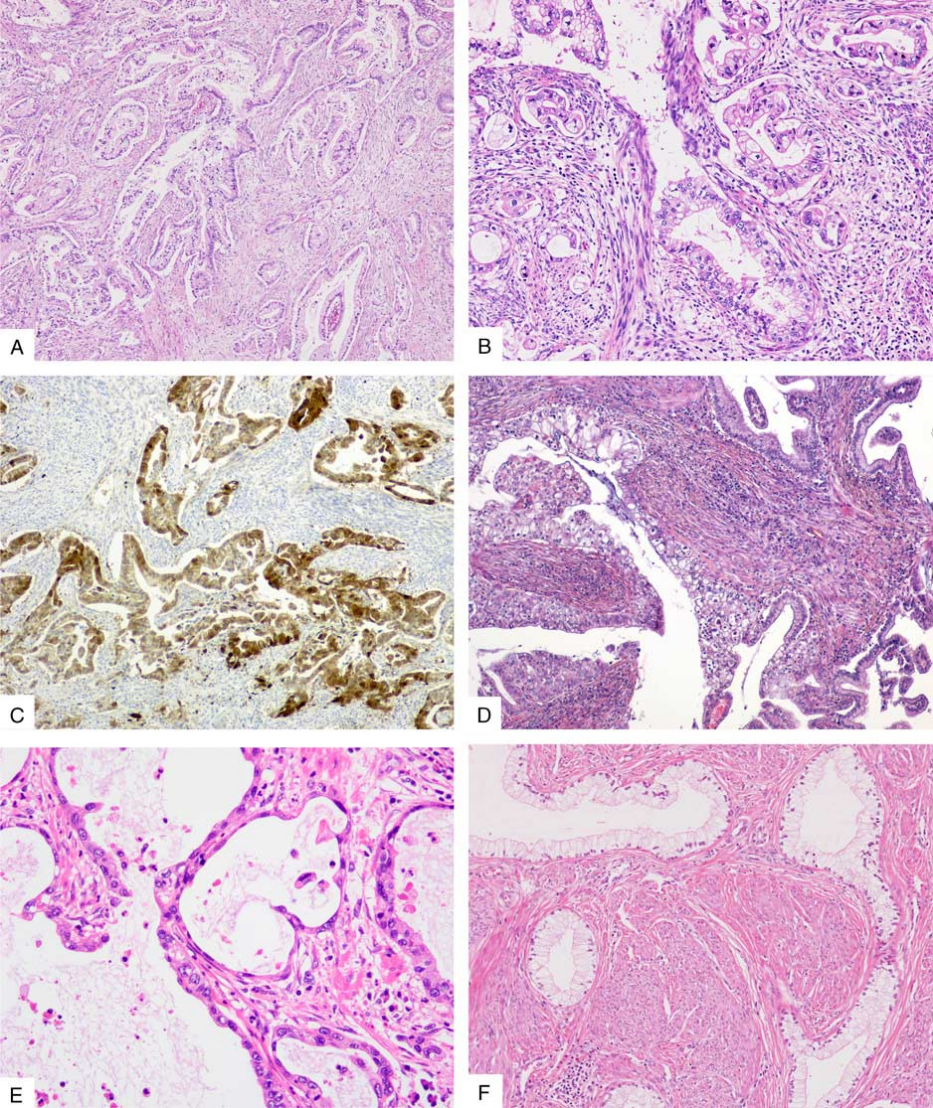
Gastric-type adenocarcinoma, human papillomavirus–independent. Typical appearance (A, B) with destructive stromal invasion by glands lined by high columnar cells with pink-to-clear cytoplasm and prominent “plant-cell-like” cytoplasmic borders. (C) Positive but non–block-type staining with p16. (D) Gastric-type adenocarcinoma with a focal component of clear cells, (E) cystic glands, (F) highly differentiated gastric-type adenocarcinoma, a term that is synonymous with “minimal deviation adenocarcinoma of mucinous type” and “adenoma malignum.”

In contrast to usual-type tumors, which typically contain elongated and pseudostratified hyperchromatic nuclei, gastric-type tumors are characterized by basally located rounded nuclei when well differentiated. The nuclei have clear or delicate, diffuse chromatin, sometimes with a distinct nucleolus, and typically appear pale in comparison to the hyperchromatic nuclei of usual-type tumors. Atypia ranges from minimal to marked. Architecturally, the tumor forms glands or solid areas, but papillary, trabecular, or single-cell patterns can be present. The glands vary in size and shape from small, simple tubular forms to cystically dilated with irregular contours and sometimes intraluminal papillary infoldings. The glands infiltrate the stroma, eliciting a desmoplastic response, and lymphovascular invasion is common. Minimal deviation adenocarcinoma of mucinous type (ie, adenoma malignum), which is included in the spectrum of gastric-type endocervical adenocarcinomas, is characterized by low-grade morphology, with very well-differentiated glands with a “claw-like” pattern, lined by cells with abundant intracytoplasmic mucin and minimally atypical nuclei. The glands are distributed haphazardly within the stroma, sometimes with minimal or no desmoplasia (Fig. 5). The terms “minimal deviation adenocarcinoma” and “adenoma malignum” are no longer recommended.

Gastric-type endocervical adenocarcinoma produces neutral gastric (pyloric-type) mucin that stains magenta (pink/red) on PAS/Alcian blue histochemical stain, whereas normal endocervical mucins are acidic and stain dark blue. As previously mentioned, gastric-type endocervical adenocarcinomas morphologically resemble adenocarcinomas of the pancreatobiliary tract, with a similar immunohistochemical profile; one difference is that most, but not all, gastric-type endocervical adenocarcinomas express PAX8 (see below) 47.

As stated previously, gastric-type endocervical adenocarcinoma is usually negative or exhibits patchy, non–block-type immunoreactivity with p16 and is HPV-negative; however, occasional examples exhibit block-type immunoreactivity with p16 10,13,47. These carcinomas exhibit aberrant (mutation-type) expression of p53 in up to 50% of cases 28. ER, PR, vimentin, p63, p40, and AR are usually negative 28,47. PAX8 is positive in 68% to 80% of gastric-type adenocarcinomas, which is useful in distinguishing these tumors from adenocarcinomas of gastrointestinal or pancreatobiliary origin. SATB2 is usually negative, although there can be weak positivity 28,47. PAX2 is typically negative. Gastric-type mucin markers, such as MUC6 and HIK1083, are positive in 60% to 80% of these carcinomas 28,43,47. Unfortunately, HIK1083 testing is not available outside of Japan (with few exceptions), and MUC6 may be expressed in other tumor types 48. Gastric-type endocervical adenocarcinomas are positive for Trefoil Factor 2 (TFF2), CK7, CEA, and CAIX and up to 50% can be positive for CK20 and CDX2 28,47. Of interest, HNF1beta can be positive in up to 90% of cases whereas Napsin A may be positive in a much smaller percentage of cases 28,49; this is important as the differential diagnosis may include clear cell carcinoma.

When gastric-type endocervical adenocarcinoma is difficult to distinguish from usual-type endocervical adenocarcinoma, the presence of prominent mitotic and apoptotic activity favors a usual-type diagnosis. MUC6, HIK1083, p16, and HPV testing may be of value. High-grade squamous intraepithelial lesions and HPV-associated AIS will favor a usual-type diagnosis, although small numbers of gastric-type endocervical adenocarcinomas may contain such lesions coincidentally. Atypical LEGH or gAIS will favor a gastric-type diagnosis. Of interest, carcinomas exhibiting overlapping morphology with areas resembling both usual-type (with prominent apoptosis and mitoses) and gastric-type tumors have been described. In these difficult cases, HPV and p16 testing can usually identify whether it is an HPV-associated mucinous carcinoma or gastric-type tumor with morphologic mimicry rather than a true mixed carcinoma, which likely does not occur 50.

Gastric-type adenocarcinoma may be particularly difficult to differentiate from clear cell carcinoma, especially with biopsy material. HNF1beta and Napsin A can be positive in both tumors, but HNF1beta is particularly likely to be positive in gastric-type endocervical adenocarcinomas, as discussed previously 49. A combination of HIK1083 and TFF2 testing can be useful, as TFF2 is expressed in 80% of gastric-type tumors compared with 12% of non–gastric-type tumors, with no clear cell carcinomas having been shown to be positive. Dual positivity for TFF2 and HIK1083 is highly specific for gastric-type endocervical adenocarcinomas 51.

When distinguishing between morphologically well-differentiated variants of gastric-type endocervical adenocarcinoma (adenoma malignum) and a variety of benign cervical glandular lesions, such as deep Nabothian cysts or endocervical glandular hyperplasia, ER/PR staining can be useful, as gastric-type tumors are usually negative and most benign glandular lesions, with the exception of LEGH and mesonephric remnants, are positive for these markers. The “claw-like” shape and deep placement of glands along with at least mild nuclear atypia and the presence of at least focal stromal desmoplasia are also more typical of gastric-type endocervical adenocarcinomas. These features are helpful in differentiating gastric-type endocervical adenocarcinoma from LEGH, in which a preserved lobular architecture with minimal cytologic atypia is observed. Immunohistochemistry is of limited value in distinguishing between LEGH and well-differentiated variants of gastric-type endocervical adenocarcinoma, emphasizing the value of H&E morphology to separate these entitites. Some authors have suggested that smooth muscle actin staining may be useful, as this marker is positive in cervical stromal cells adjacent to invasive glands of gastric-type endocervical adenocarcinoma and negative in the stromal cells surrounding LEGH 18. Gastric-type tumors, including well-differentiated variants, exhibit aberrant/mutation p53 staining in ~50% of cases, whereas LEGH exhibits wild-type immunoreactivity. PAX2 is negative in gastric-type endocervical adenocarcinoma and positive in LEGH 47,52.

Theoretically, in all gastric-type endocervical adenocarcinomas, a metastasis, especially from the pancreas or biliary tree, should be ruled out by clinical means, as the morphology and the immunophenotype of gastric-type endocervical adenocarcinomas and pancreatic/biliary adenocarcinomas can be very similar, if not identical. PAX8 positivity favors a primary cervical tumor diagnosis, but not all gastric-type endocervical adenocarcinomas of the cervix are PAX8-positive. The differential diagnosis with clear cell carcinoma is discussed later in the text.

The molecular mechanisms underlying the pathogenesis of gastric-type endocervical adenocarcinoma are still emerging. These tumors sometimes develop in patients with Peutz-Jeghers syndrome, an autosomal dominant disorder caused by germline mutation of *STK11*. Somatic mutations of *STK11* have also been identified in some sporadic cases 53–57. Recent molecular studies have shown genetic alterations, such as somatic mutations in *TP53*, *CDKN2A*, and *ERBB2/ERBB3*, and less commonly, mutations in *GNAS*, *SMAD4*, and *PIK3CA* 56–60.

### Clear Cell Type Endocervical Adenocarcinoma

This rare tumor of the cervix, which accounts for ~4% of all cervical adenocarcinomas, occurs in young women exposed to diethylstilbestrol in utero (when the tumor usually involves the ectocervix) and sporadically in young or older postmenopausal women (when the tumor predominantly involves the endocervix) 61. Cases associated with diethylstilbestrol are rare nowadays.

### Issues Regarding Clear Cell Type

- The precursor lesion of cervical clear cell carcinoma is still unknown.
- The tumor cells can have clear or eosinophilic cytoplasm, which may mimic gastric-type tumors.
- Clear cell carcinomas often show patchy staining with p16, although only rare cases have block-type staining.
- HNF1beta is a nonspecific marker, as it is positive in many other types of endocervical adenocarcinoma.
- The main malignant differential diagnoses are gastric-type and mesonephric-type endocervical adenocarcinomas.
- Before diagnosing a primary cervical clear cell carcinoma, an endometrial primary should be excluded.
- Clear cell carcinoma can also mimic benign glandular lesions, such as microglandular hyperplasia and Arias-Stella reaction.

**FIG. 6:**
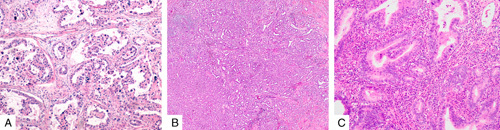
Other human papillomavirus-independent adenocarcinomas: (A) clear cell, (B) mesonephric, and (C) endometrioid.

Similar to clear cell carcinomas of the uterine corpus or ovary, clear cell carcinoma of the cervix is characterized by solid, papillary, and/or tubulocystic architecture with polygonal or hobnail cells with abundant clear or eosinophilic cytoplasm. A tubulocystic architecture is especially common in primary cervical clear cell carcinoma, and a hyalinized stroma is often present. Mitotic activity is often, but not always, low (Fig. 6).

Clear cell carcinomas are negative for HPV and usually exhibit negative or focal p16 staining but can occasionally demonstrate block-type positivity 13. CK7 and PAX8 are positive in most cases, whereas CDX2, ER, PR, vimentin, TFF2, TTF1, MUC6, HIK1083, CAIX, p63, p40, HER2, GATA3, and AR are usually negative. P53 can exhibit aberrant/mutation-type staining in up to 15% of carcinomas 28. HNF1beta and Napsin A are only positive in ~40% to 70% of cases 28,49.

The differential diagnosis of clear cell endocervical adenocarcinoma includes gastric-type and mesonephric-type endocervical adenocarcinomas, and useful morphologic features and ancillary tests to differentiate clear cell from gastric-type carcinomas have been detailed above. To summarize, although both clear cell carcinoma and gastric-type adenocarcinomas typically have low nuclear to cytoplasmic ratios with prominent cytoplasmic borders, the absence of characteristic clear cell carcinoma growth patterns and presence of obvious intracytoplasmic mucin would be more in keeping with a diagnosis of gastric-type adenocarcinoma. Furthermore MUC6, HIK1083, and carbonic anhydrase IX are significantly more commonly positive in gastric-type adenocarcinoma, whereas Napsin A positivity will favor a clear cell carcinoma diagnosis. None of these markers are totally specific. It is important to remember that these tumors are HPV-independent. Clear cell carcinomas can also mimic benign glandular lesions, such as Arias-Stella reaction and microglandular hyperplasia.

Microsatellite instability has been reported in cervical clear cell carcinomas, whereas no mutations in *KRAS*, *HRAS*, or *TP53* were found on molecular analysis in an older study 62. More recent data are not available.

### Mesonephric-type Endocervical Adenocarcinoma

Mesonephric endocervical adenocarcinoma of the cervix is very rare, accounting for <1% of all cervical adenocarcinomas and originating from mesonephric remnants deep in the lateral cervical wall 13.

### Issues Regarding Mesonephric-type Endocervical Adenocarcinomas

- A well-defined precursor lesion has not been described, although some mesonephric adenocarcinomas likely arise from mesonephric remnants/hyperplasia.
- Characteristically, these tumors exhibit an admixture of various architectural growth patterns.
- The tumor cells generally have scant cytoplasm and atypical nuclei that may resemble those of papillary thyroid carcinomas.
- These tumors can mimic endometrioid or serous carcinoma, clear cell carcinoma, or usual-type HPV-associated tumors, and ancillary tests can assist in their differentiation.
- A spindle-cell component can sometimes be seen; a diagnosis of carcinosarcoma should be reserved for those rare tumors with heterologous elements.

Morphologically, these tumors characteristically exhibit an admixture of growth patterns, including ductal, tubular, glomeruloid, papillary, retiform, solid, sex cord-like, and spindled (sarcomatoid). A small tubular pattern, sometimes containing intraluminal eosinophilic colloid-like material, is especially characteristic. The tumor cells usually have scant cytoplasm, and many nuclei may be oval, optically clear, grooved, and overlapping, similar to papillary thyroid carcinoma. Other nuclei are hyperchromatic. Adjacent benign mesonephric remnants are identified in a minority of cases. The tumor usually develops in the lateral aspect of the cervix, but when discovered, this origin in the lateral walls is often not apparent with circumferential cervical involvement (Fig. 6).

These tumors are negative for HPV, and are usually negative (or exhibit non–block-type positivity) for p16. ER and PR tend to be completely negative 13. GATA3 and PAX8 are frequently positive, while Calretinin, CD10, HNF1beta, and TTF1 are often, but not always, positive 63–67.

An important differential diagnostic consideration is the usual-type endocervical adenocarcinoma. HPV testing, p16, and GATA3 immunohistochemistry can be diagnostically useful in these cases, as mesonephric endocervical adenocarcinoma will be HPV-negative and p16-negative (or non–block-type) and typically positive for GATA3.

In contrast to mesonephric-type tumors, endometrioid adenocarcinomas of the endometrium are mostly ER-positive and PR-positive, GATA3-negative and TTF1-negative, and show confirmatory endometrioid features 28. Mesonephric carcinomas rarely form papillae lined by a monolayer of low-columnar mildly atypical cells, like clear cell carcinoma. Mesonephric carcinomas with a focal papillary architecture will almost always display typical growth patterns of mesonephric carcinomas in other portions of the tumor, facilitating its distinction from clear cell carcinoma. Clear cell carcinomas are, from a practical standpoint, negative for the “confirmatory” mesonephric markers, GATA3, and TTF1.

Florid mesonephric hyperplasia can mimic mesonephric adenocarcinoma, but it usually does not form a mass lesion. Nuclear atypia, mitotic activity, morphologic patterns other than small tubular, a stromal reaction, lymphovascular invasion, and spread outside the cervix favor a mesonephric-type adenocarcinoma diagnosis.

Recent molecular studies have demonstrated *KRAS* mutations in most mesonephric adenocarcinomas of the cervix, whereas a smaller number show activating *NRAS* mutations. Demonstration of a *KRAS* or *NRAS* mutation may be useful in diagnosing a mesonephric adenocarcinoma and distinguishing this from florid mesonephric hyperplasia, which does not exhibit mutations 68. Mutations in *ARID1A/B* and chromosomal abnormalities with copy number gains in 1q, loss of 1p, and gain of chromosomes 10 and 12 have also been reported 68,69.

### Endometrioid-type Endocervical Adenocarcinoma

This is a very rare primary tumor of the cervix that is said to arise in the setting of endometriosis. An endometrioid-type endocervical adenocarcinoma diagnosis should be made with caution; previously, in many institutions, a diagnosis of primary endometrioid adenocarcinoma of the cervix was often made for usual-type adenocarcinomas with minimal intracytoplasmic mucin. When strict diagnostic criteria are used, this is an extremely rare tumor, accounting for <1% of cervical carcinomas 13.

### Issues Regarding Endometrioid-type Endocervical Adenocarcinomas

- Primary cervical endometrioid-type endocervical adenocarcinomas are very rare, and tumors of the cervix with an endometrioid appearance are almost all HPV-positive, with most cases being of usual type.
- Cervical endometrioid adenocarcinoma is thought to arise in the setting of endometriosis; however, a premalignant lesion is not well defined.
- Before diagnosing a primary cervical endometrioid adenocarcinoma, spread from the corpus should be excluded.
- The immunohistochemical profile is expected to be similar to that of endometrioid adenocarcinoma of the uterine corpus.

According to IECC/WHO 2020, endometrioid adenocarcinoma of the cervix must display endometrioid morphology with “confirmatory endometrioid features,” such as: (a) at least focally identified low-grade endometrioid glands, (b) lined by columnar cells, (c) pseudostratified nuclei, (d) no more than moderate atypia, (e) with or without squamous differentiation, (f) and/or endometriosis present, and (g) lacking HPV-associated features, such as prominent mitotic figures and apoptotic bodies (Fig. 6) 13.

p16 is usually negative or non–block-type; HPV is negative 13. ER, PR, vimentin, CK7, and PAX8 are usually positive. Aberrant/mutation-type p53 and positivity for MUC 6 and SATB2 occur in one-third of cases. p63, p40, HER2, AR, GATA3, HIK1083, HNF1beta, Napsin A, CK20, and TTF1 are negative 28.

The main differential diagnostic consideration is usual-type endocervical adenocarcinoma and, as mentioned above, a reflexive diagnosis of endometrioid-type endocervical adenocarcinoma should not be made in the presence of a mucin-depleted adenocarcinoma. The other main differential diagnostic consideration is an endometrioid adenocarcinoma arising from the corpus or ovary, with spread into the cervix. The differential diagnosis can be difficult on a biopsy with minimal tissue, as the morphology and immunohistochemical profiles of endometrioid carcinomas arising at these sites are identical, and HPV is negative. Correlation with clinical, radiologic, and macroscopic features is essential, as their management depends largely on the site of origin.

### Adenocarcinoma NOS-type Endocervical Adenocarcinoma

These HPV-independent tumors cannot be classified into one of the other types (Fig. 4A). Morphologically, they are poorly differentiated tumors with predominantly solid architecture and highly atypical nuclei, whereas the amount of intracytoplasmic mucin is typically minimal. p16 is negative or non–block-type, and HPV is negative. This diagnosis should be rarely made.

### Recommendations for the Diagnosis of HPV-independent Endocervical Adenocarcinomas

- Immunohistochemistry is of limited value in distinguishing between LEGH and well-differentiated variants of gastric-type endocervical adenocarcinoma, and this is a predominantly morphologic diagnosis.
- Serous carcinoma of the cervix does not exist.
- True endometrioid adenocarcinoma of the endocervix is extremely rare and should not be reflexively diagnosed in the presence of a mucin-poor endocervical adenocarcinoma.
- Immunohistochemical markers are useful for differentiating between the various HPV-independent histologic types, and HPV testing for differentiating between HPV-associated and -independent endocervical adenocarcinomas.

## CONCLUSIONS

Endocervical adenocarcinomas represent a very heterogeneous group of tumors. Usual and mucinous types are associated with HPV infection; gastric, clear cell, mesonephric, and endometrioid types are not driven by HPV. The distinction between HPV-associated and HPV-independent endocervical adenocarcinomas has important clinical implications, as HPV-independent tumors tend to develop in older patients, present at a higher clinical stage, have a worse prognosis, have different and unusual patterns of spread, and respond differently to typical oncologic treatment for cervical cancer. Moreover, the fact that some endocervical adenocarcinomas are HPV-independent is important for HPV vaccination strategies and HPV-based screening programs.

As HPV-associated and HPV-independent endocervical adenocarcinomas have different molecular drivers, it is imperative to understand their unique features, which is necessary for the development of future targeted therapies, ongoing genomic studies, and clinical trials, which hopefully will add more to our understanding of this uncommon group of tumors.
